# Physiologic pacing in congenitally corrected transposition of the great arteries with electroanatomic mapping guidance: a case report

**DOI:** 10.1093/ehjcr/ytae520

**Published:** 2024-09-23

**Authors:** Ofir Brem, Kirill Buturlin, Shimon Kolker, Nili Schamroth Pravda

**Affiliations:** Faculty of Medicine, Tel Aviv University, PO Box 39040, Klatzkin 35, Tel Aviv 6997801, Israel; Faculty of Medicine, Tel Aviv University, PO Box 39040, Klatzkin 35, Tel Aviv 6997801, Israel; Department of Cardiology, Rabin Medical Center, Zeev Jabotinsky 39, Petah Tikva 4941492, Israel; Faculty of Medicine, Tel Aviv University, PO Box 39040, Klatzkin 35, Tel Aviv 6997801, Israel; Department of Cardiology, Rabin Medical Center, Zeev Jabotinsky 39, Petah Tikva 4941492, Israel; Faculty of Medicine, Tel Aviv University, PO Box 39040, Klatzkin 35, Tel Aviv 6997801, Israel; Department of Cardiology, Rabin Medical Center, Zeev Jabotinsky 39, Petah Tikva 4941492, Israel

**Keywords:** Case report, Cardiac pacemaker, Transposition of the great arteries, Congenital heart defect, Electro-anatomical mapping, Cardiac resynchronization therapy

## Abstract

**Background:**

This case report details the application of left bundle branch pacing in a patient with congenitally corrected transposition of the great arteries (cc-TGA), a rare congenital heart defect characterized by anatomical complexities that pose unique challenges in the management of device-related complications and heart failure. The patient’s history is notable for complex anatomical considerations, cardiovascular implantable electronic device (CIED) infection, and heart failure.

**Case summary:**

The patient underwent a series of interventions, including treatment for pocket-site infections, abandonment of epicardial leads, and an unsuccessful attempt at trans-catheter leadless pacemaker implantation. Given the patient’s complex anatomy and prior CIED infection, traditional pacing methods were deemed unsuitable, leading to the selection of left bundle branch pacing. The lead implantation was guided using 3D electro-anatomical mapping to ensure synchronous physiologic pacing in a patient with heart failure.

**Discussion:**

The case underscores the heightened risks faced by cc-TGA patients, with a focus on systemic right ventricular dysfunction and pacing-induced ventricular dysfunction. In these patients, ventricular synchrony is critical and can be achieved with biventricular pacing. Physiologic pacing emerges as a promising alternative to cardiac resynchronization therapy (CRT), especially in cases where endovascular CRT is unfeasible. This case demonstrates the utilization of 3D electro-anatomical mapping for achieving successful physiologic pacing in complex congenital heart lesions. At the 12-month follow-up, the patient presented with stable clinical status and a narrow QRS complex. Echocardiography indicated improvement in the right systemic ventricular function.

**Learning points**

- To explore the utility and challenges of physiologic pacing in patients with complex anatomical considerations and congenital heart disease.
- To highlight the use of 3D electro-anatomical mapping in the context of left bundle branch pacing, assessing its utility in guiding lead placement and optimizing pacing outcomes.
- This case highlights the harm that cardiovascular implantable electronic device infections can inflict and the clinical sequelae of these infections.

## Introduction

Patients with congenitally corrected transposition of the great arteries (cc-TGA) often require lifelong, intricate medical care due to the unique challenges posed by their condition.^1^ Advancements in pacing techniques are critical for preserving systemic ventricular function in complex congenital heart defects such as cc-TGA.^2^ This case report explores the journey of a 26-year-old male patient with cc-TGA, complicated by extensive pacemaker infection and cardiac dyssynchrony, requiring advanced pacing techniques to restore an acceptable quality of life. Our narrative displays the balance between advanced interventional procedures and the management of postoperative complications. In this case, we highlight the use of 3D electro-anatomical mapping, a cutting-edge technology that allows precise navigation of the heart’s conduction system, to guide conduction system pacing for the management of systemic ventricular dysfunction in cc-TGA.

## Summary figure

Case figure. Timeline of events related to the care of the patient from childhood.

**Figure:**
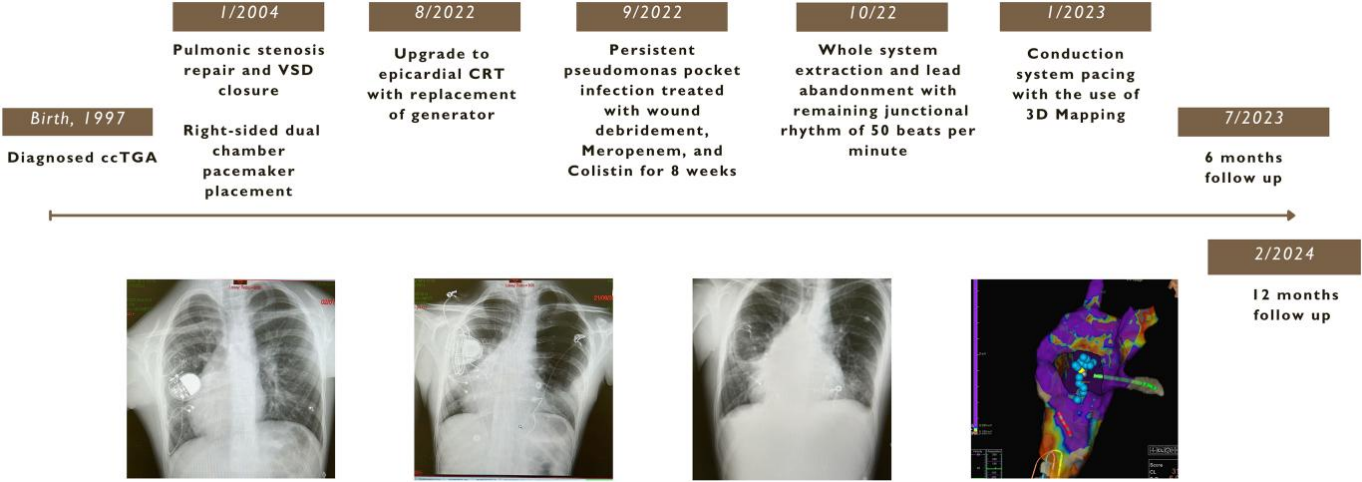


## Case presentation

This case report details the medical journey of a 26-year-old male with cc-TGA, dextrocardia, and situs solitus, complicated by an extensive pacemaker infection and resultant cardiac dyssynchrony.

The patient’s complex history begins with repair of pulmonic stenosis with a pulmonary homograft implantation and VSD closure using a patch in childhood. The procedure was complicated by complete atrioventricular block, and an epicardial pacemaker was implanted. Subsequently, he was admitted with fever and chills, due to a pocket infection of his pacemaker. Despite prolonged intravenous course of antibiotics, the patient had persistent growth of *Pseudomonas* spp. from the wound.

**Figure 1:**
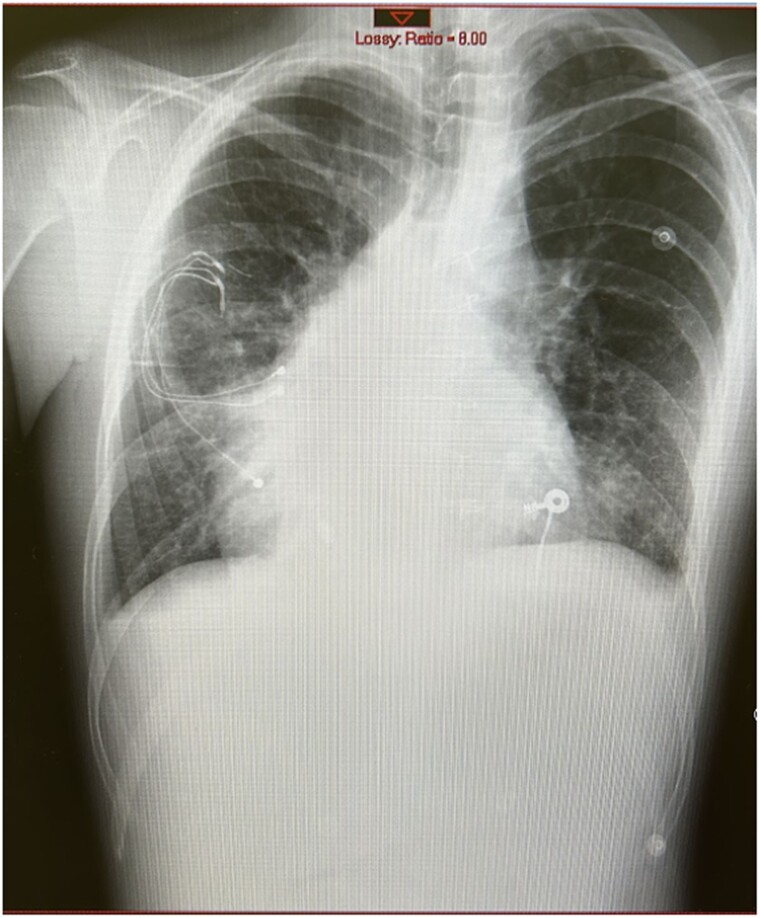
Anterior-to-posterior chest X-ray showing dextrocardia and abandoned epicardial leads of the cardiac resynchronization device.

In the years following initial pacemaker placement, the patient developed clinical heart failure with global reduction in the function of the systemic right ventricle and an ejection fraction of 35%. Pacing-induced ventricular dyssynchrony was thought to be a considerable factor exacerbating the heart failure.^3^ Consequently, the patient was upgraded to an epicardial lead cardiac resynchronization therapy (CRT) with replacement of the original generator. Soon after CRT implantation, the patient presented to the emergency room with fevers and chills, associated with erythema and pain over the site of the CRT device. The patient had negative blood cultures, negative modified duke criterion, and no evidence endocarditis or another source of infection. The working diagnosis at this point was a pocket-site infection, treated with wound debridement and extended antibiotic course including both meropenem and colistin. Due to persistent wound infection, the distal ends of the epicardial leads were cut and abandoned (*Figure 1*).

Following lead abandonment, the infection subsided, but the patient remained pacemaker-dependent in a junctional rhythm of 50 b.p.m. A Micra™ (Medtronic) trans-catheter leadless pacemaker was unsuccessfully attempted in the right-sided, morphological left ventricle.

Cardiac resynchronization therapy was preferred due to prior pacemaker-induced cardiomyopathy. Given the known anatomical variations in cc-TGA, cardiac gated computed tomography (CT) was performed and revealed anatomical narrowing of the coronary sinus.^3^ Narrowing of the coronary sinus made endovascular CRT poorly feasible, prompting the selection of endovascular left bundle branch physiologic pacing.^4^ Given the anomalous anatomy, 3D anatomical-electric mapping was utilized to identify the most proximal portion of the His bundle and initiate physiologic pacing at the anterior and posterior fascicles of the left bundle branch.

**Figure 2:**
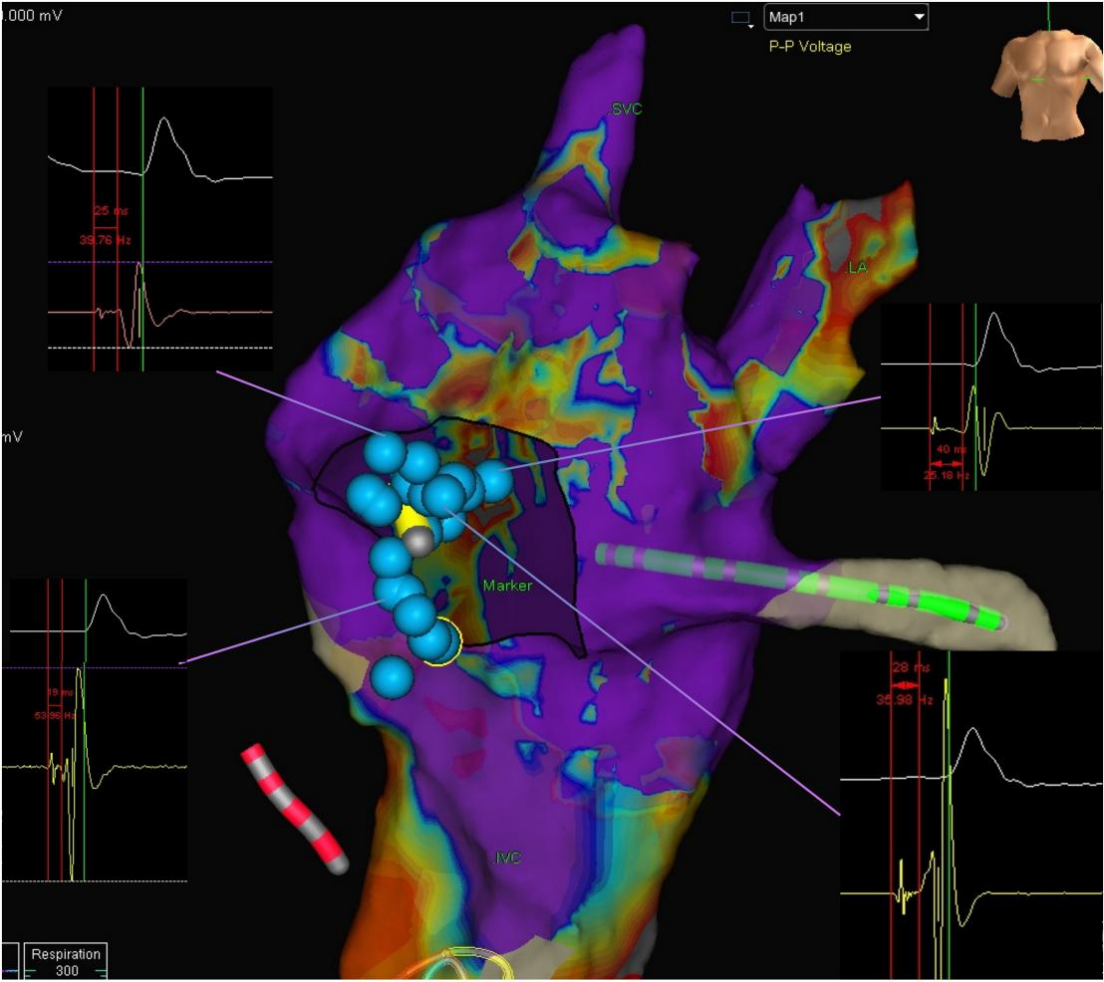
Anterior-to-posterior view of the sub-pulmonic ventricle with selected fascicular signals guiding the implantation of conduction system pacing.

**Figure 3:**
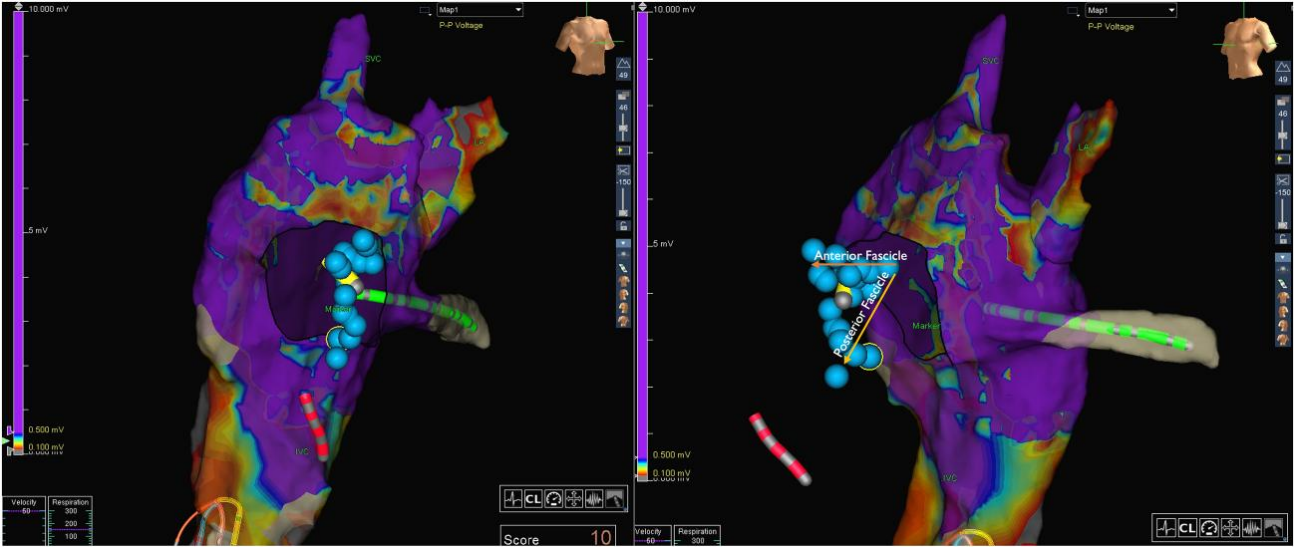
Dual-pane 3D electro-anatomical voltage map of the left, sub-pulmonic ventricle in the right anterior oblique/left anterior oblique views. Arrows indicate the left bundle branches. The superior arrow aligns with the anterior left bundle branch. The inferior arrow aligns to the posterior left bundle branch. The yellow catheter shadow aligns with the anatomical location of conduction system pacing lead placement.

**Figure 4:**
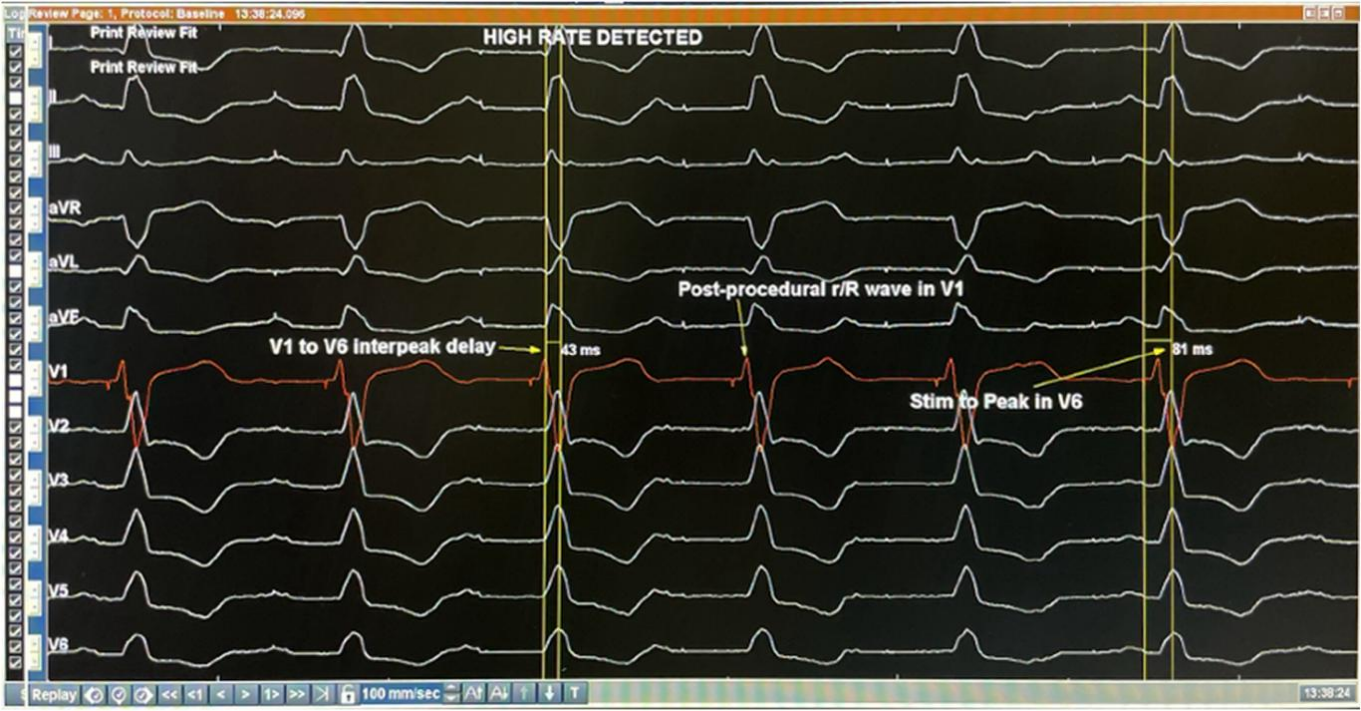
Post-procedural 12-lead electrocardiogram displaying r/R wave in lead V1 and a stim to peak delay of 81 ms in lead V6. Precordial V1–V6 displays an inter-peak delay of 43 ms.

In the procedure, the HD Grid Mapping catheter, RV and CS catheter were used along with the EnSite™ precision mapping system (Abbott) to construct a 3D map of the left, sub-pulmonic, ventricle. The left anterior and left posterior branches, located on the right side of ventricular septum, were labelled according to the distance from the His deflection (*Figures 2* and *3*). Prior to lead implantation, pacing of the left bundle branch produced a r/R wave in lead V1, with V1-V6 inter-peak delay of 43 ms, and stim to peak duration of 81 ms in V6 (*Figure 4*). Afterwards, the 3830 Selectsecure Medtronic lead was screwed successfully into the left anterior branch area under 3D map guidance with a final QRS duration reduction from 110 to 100 ms. The lead parameters following implantation were stable. The ventricular lead had a threshold of 1.2 volts, an impedance of 673 Ohm and a p/R wave of 7.1 mv.

**Figure 5:**
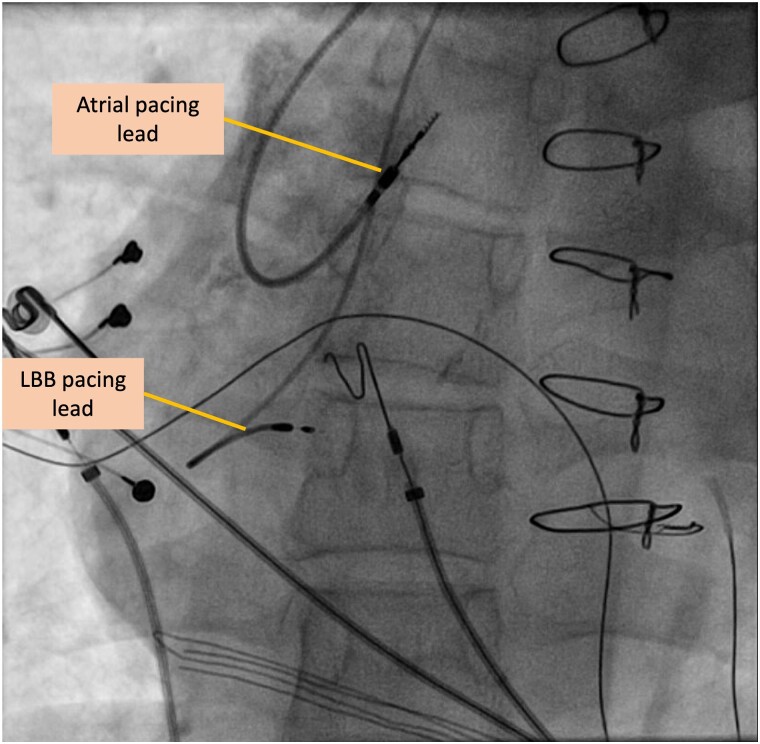
Anterior-to-posterior fluoroscopy view of the pacing leads in the atrial and left bundle branch position.

In summary, this patient with cc-TGA had extensive cardiovascular implantable electronic device (CIED) infection and a history of pacing-induced reduction of ventricular function. Left bundle branch pacing, with 3D electro-anatomical guidance, allowed for successful ventricular synchrony and pacing. Given the anatomical constraints, conduction system pacing was appropriately utilized as an alternative pacing method to CRT (*Figure 5*).

At 12-month follow-up, the patient remained clinically well, exhibiting consistent pacemaker variables and a narrow QRS complex. Follow-up echocardiography demonstrated improvement in the systemic ventricular ejection fraction which was only mildly reduced. The data on long-term outcomes of physiologic pacing in patients with cc-TGA is not yet established.^5^ Thus, continued follow-up of ventricular function and pacemaker is vital.^6^

## Discussion

Patients with cc-TGA are at increased risk of congenital heart block, and the function of the systemic right ventricle dictates their prognosis.^1^ As a result, cardiac dyssynchrony and pacing-induced ventricular dysfunction can be detrimental to the care of patients with cc-TGA.^7^ Biventricular pacing is believed to prevent, and sometimes restore, this pacemaker-induced loss of ventricular function.^5,8,9^

Physiologic pacing is a currently evolving technique that is non-inferior to CRT in terms of ejection fraction improvement, procedural complexity, effect on mortality, and heart failure hospitalization.^10–12^ Physiologic pacing is achieved by implantation of a pacing lead in the distal His bundle and/or proximal left bundle branches.^13^

In patients with cc-TGA, the left ventricle is in the sub-pulmonary position, and the outflow tract extends to the origin to the pulmonary artery. In this anomaly, the absence of the pectinate muscle and relatively smooth walls of this, structurally left, ventricle made the insertion and stabilization of leadless pacemaker systems extremely difficult. Similarly, endovascular CRT was impractical given the coronary sinus narrowing demonstrated on CT.

In contrast, the structural defect in cc-TGA allows the distal His and left bundle branches to be directly accessible from a right-sided transvenous approach, thus allowing the left bundle branch to be targeted without the need for deep intra-septal penetration.^14^ 3D electro-anatomical mapping was utilized to visualize the left bundle branch for precise implantation of the lead. This case serves as an example of the complex electrophysiological management of patients with ACHD and the utility of advanced electro-anatomical guidance in optimizing patient care.

Limited literature exists on this method, yet it has been demonstrated as feasible in a retrospective registry by Moore *et al*. for the Pediatric and Congenital Electrophysiology Society (PACES) and the International Society for Adult Congenital Heart Disease (ISACHD), where 13 of 15 patients with cc-TGA attained successful permanent conduction system pacing. Of these, 9 cases benefited from the assistance of electromagnetic mapping. No complications were reported, and a significant reduction in QRS duration was observed compared to baseline ventricular pacing.^14^ Additionally, a case documented by Becker *et al*.^15^ presented similar outcomes in conduction system pacing post-double switch surgery in a patient with cc-TGA.^15^ The outcomes observed in these studies, and our own, point to the potential efficacy and feasibility of conduction system pacing for patients with cc-TGA and systemic ventricular dysfunction related to univentricular pacing.

While the applications of physiologic pacing in ACHD are exciting, the longevity of the pacemaker remains a concern for long-term outcomes. These preliminary findings underscore the necessity for further investigative studies across larger patient populations to evaluate the benefits and potential complications of physiologic pacing in complex congenital heart lesions. Data on the long-term outcomes of physiologic pacing within ACHD is still sparse and necessary for the objective evaluation of this treatment.

## Conclusions

This case report demonstrates that physiologic pacing can be successfully achieved using 3D electro-anatomical mapping in patients with complex congenital heart lesions. This innovative approach may provide clinical benefits for a broad range of patients, including those with complex anatomical lesions. Further long-term data are needed to evaluate the feasibility across diverse anatomical structures and the long-term durability of this pacing method.

## Lead author biography


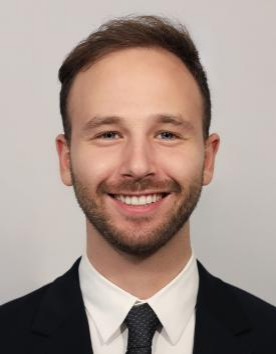


Ofir Brem, B.A., is currently a third year medical student studying at Tel Aviv University and pursuing congenital heart disease research at the Rabin Medical Center under the guidance of Dr Nili Schamroth Pravda.

## Data Availability

The data will be shared on reasonable request to the corresponding author.

## Notes

**Consent:** Consent for submission and publication of this case report, including images and associated text, has been obtained from the patient in line with COPE guidelines.

**Funding:** No funding to declare.

## References

[1] Graham TP Jr , Bernard YD, Mellen BG, Celermajer D, Baumgartner H, Cetta F, et al Long-term outcome in congenitally corrected transposition of the great arteries. J Am Coll Cardiol 2000;36:255–261. 10898443 10.1016/s0735-1097(00)00682-3

[2] Stout KK , Daniels CJ, Aboulhosn JA, Bozkurt B, Broberg CS, Colman JM, et al 2018 AHA/ACC guideline for the management of adults with congenital heart disease: executive summary: a report of the American College of Cardiology/American Heart Association Task Force on Clinical Practice Guidelines. J Am Coll Cardiol 2019;73:1494–1563. 30121240 10.1016/j.jacc.2018.08.1028

[3] Bottega NA , Kapa S, Edwards WD, Connolly HM, Munger TM, Warnes CA, et al The cardiac veins in congenitally corrected transposition of the great arteries: delivery options for cardiac devices. Heart Rhythm 2009;6:1450–1456. 19968924 10.1016/j.hrthm.2009.07.037

[4] Moore JP , Cho D, Lin JP, Lluri G, Reardon LC, Aboulhosn JA, et al Implantation techniques and outcomes after cardiac resynchronization therapy for congenitally corrected transposition of the great arteries. Heart Rhythm 2018;15:1808–1815. 30125719 10.1016/j.hrthm.2018.08.017

[5] Hofferberth SC , Alexander ME, Mah DY, Bautista-Hernandez V, del Nido PJ, Fynn-Thompson F. Impact of pacing on systemic ventricular function in L-transposition of the great arteries. J Thorac Cardiovasc Surg 2016;151:131–138. 26410005 10.1016/j.jtcvs.2015.08.064

[6] Vijayaraman P , Naperkowski A, Subzposh FA, Abdelrahman M, Sharma PS, Oren JW, et al Permanent His-bundle pacing: long-term lead performance and clinical outcomes. Heart Rhythm 2018;15:696–702. 29274474 10.1016/j.hrthm.2017.12.022

[7] Yeo WT , Jarman JWE, Li W, Gatzoulis MA, Wong T. Adverse impact of chronic subpulmonary left ventricular pacing on systemic right ventricular function in patients with congenitally corrected transposition of the great arteries. Int J Cardiol 2014;171:184–191. 24374205 10.1016/j.ijcard.2013.11.128

[8] Dubin AM , Janousek J, Rhee E, Strieper MJ, Cecchin F, Law IH, et al Resynchronization therapy in pediatric and congenital heart disease patients: an international multicenter study. J Am Coll Cardiol 2005;46:2277–2283. 16360058 10.1016/j.jacc.2005.05.096

[9] Janousek J , Tomek V, Chaloupecký VA, Reich O, Gebauer RA, Kautzner J, et al Cardiac resynchronization therapy: a novel adjunct to the treatment and prevention of systemic right ventricular failure. J Am Coll Cardiol 2004;44:1927–1931. 15519030 10.1016/j.jacc.2004.08.044

[10] Wang Y , Zhu H, Hou X, Wang Z, Zou F, Qian Z, et al Randomized trial of left bundle branch vs biventricular pacing for cardiac resynchronization therapy. J Am Coll Cardiol 2022;80:1205–1216. 36137670 10.1016/j.jacc.2022.07.019

[11] Vijayaraman P , Sharma PS, Cano Ó, Ponnusamy SS, Herweg B, Zanon F, et al Comparison of left bundle branch area pacing and biventricular pacing in candidates for resynchronization therapy. J Am Coll Cardiol 2023;82:228–241. 37220862 10.1016/j.jacc.2023.05.006

[12] Chung MK , Patton KK, Lau C-P, Dal Forno ARJ, Al-Khatib SM, Arora V, et al 2023 HRS/APHRS/LAHRS guideline on cardiac physiologic pacing for the avoidance and mitigation of heart failure. Heart Rhythm 2023;20:e17–e91. 37283271 10.1016/j.hrthm.2023.03.1538 PMC11062890

[13] Vernooy K , Keene D, Huang W, Vijayaraman P. Implant, assessment, and management of conduction system pacing. Eur Heart J Suppl 2023;25:G15–G26. 37970519 10.1093/eurheartjsupp/suad115 PMC10637838

[14] Moore JP , Gallotti R, Shannon KM, Pilcher T, Vinocur JM, Cano Ó, et al Permanent conduction system pacing for congenitally corrected transposition of the great arteries: a Pediatric and Congenital Electrophysiology Society (PACES)/International Society for Adult Congenital Heart Disease (ISACHD) Collaborative Study. Heart Rhythm 2020;17:991–997. 10.1016/j.hrthm.2020.01.033. 32243875

[15] De Becker B , O’Neill L, Pierard S, Le Polain De Waroux J-B. Cardiac resynchronization therapy using conduction system pacing after double-switch surgery for congenitally corrected transposition of the great arteries: a case report. Eur Heart J Case Rep 2023;7:ytad382. 37637094 10.1093/ehjcr/ytad382 PMC10456210

